# Transcription Factors and Methylation Drive Prognostic miRNA Dysregulation in Hepatocellular Carcinoma

**DOI:** 10.3389/fonc.2021.691115

**Published:** 2021-07-01

**Authors:** Shijie Qin, Jieyun Xu, Yunmeng Yi, Sizhu Jiang, Ping Jin, Xinyi Xia, Fei Ma

**Affiliations:** ^1^ Laboratory for Comparative Genomics and Bioinformatics & Jiangsu Key Laboratory for Biodiversity and Biotechnology, College of Life Science, Nanjing Normal University, Nanjing, China; ^2^ Institute of Laboratory Medicine, Jinling Hospital, Nanjing University School of Medicine, The First School of Clinical Medicine, Southern Medical University, Nanjing, China; ^3^ College of Arts and Sciences, Emory University, Atlanta, GA, United States

**Keywords:** hepatocellular carcinoma (HCC), miRNA, methylation, prognosis, transcription factor

## Abstract

Many dysregulated microRNAs (miRNAs) have been suggested to serve as oncogenes or tumor suppressors to act as diagnostic and prognostic factors for HCC patients. However, the dysregulated mechanisms of miRNAs in HCC remain largely unknown. Herein, we firstly identify 114 disordered mature miRNAs in HCC, 93 of them are caused by dysregulated transcription factors, and 10 of them are driven by the DNA methylation of their promoter regions. Secondly, we find that seven up-regulated miRNAs (miR-9-5p, miR-452-5p, miR-452-3p, miR-1180-3p, miR-4746-5p, miR-3677-3 and miR-4661-5p) can promote tumorigenesis *via* inhibiting multiple tumor suppressor genes participated in metabolism, which may act as oncogenes, and seven down-regulated miRNAs (miR-99-5p, miR-5589-5p, miR-5589-3p, miR-139-5p, miR-139-3p, miR-101-3p and miR-125b-5p) can suppress abnormal cell proliferation *via* suppressing a number of oncogenes involved in cancer-related pathways, which may serve as tumor suppressors. Thirdly, our findings reveal a mechanism that transcription factor and miRNA interplay can form various regulatory loops to synergistically control the occurrence and development of HCC. Finally, our results demonstrate that this key transcription factor FOXO1 can activate a certain number of tumor suppressor miRNAs to improve the survival of HCC patients, suggesting FOXO1 as an effective therapeutic target for HCC patients. Overall, our study not only reveals the dysregulated mechanisms of miRNAs in HCC, but provides several novel prognostic biomarkers and potential therapeutic targets for HCC patients.

## Introduction

Currently, the 5-year survival rate of liver cancer patients is only 20.3%, and even below 8% in underdeveloped countries or regions (1–3). Liver cancer mainly includes two major subtypes, i.e. hepatocellular carcinoma (HCC) and cholangiocarcinoma, in particular HCC is the most important subtype (about 80% of all liver cancer patients) (3). The occurrence of HCC is usually associated with virus infection (e.g. HBV or HCV), alcoholism, and metabolic syndrome (e.g. fatty liver) (4–6). Importantly, HCC can only be detected by specific markers (e.g. AFP) at this later stage due to the lack of early diagnostic markers, which leads to the lower survival of HCC patients (7–9). Therefore, the discovery of novel diagnostic and prognostic factors has very important clinical value in promoting the survival of HCC patients.

At present, studies have demonstrated that miRNAs can serve as oncogenes or tumor suppressors to regulate the occurrence and development of tumor (10–12). Many miRNAs have been reported to be closely associated with the progression of HCC (4, 6, 12). For instance, miR-16 could target *Bcl-2* to regulate the apoptotic process of HCC (13, 14), miR-21 could negatively regulate the expression of tumor suppressor genes *PTEN* and *MAP2K3* (15–17), the down-regulated miR-139 is markedly associated with the poor prognosis of HCC patients (18, 19), miR-122 could target *PI3K* and *Bcl-w* to activate the RTK survival pathway and the anti-apoptotic signaling pathway in HCC (20, 21), miR-223 could target the oncogene *C-Myc* (22), and miR-125b could target *cMet*, *MMP* and *PGF* to promote tumor angiogenesis and metastasis of HCC (23), as well as miR-34a could modulate cell cycle and inflammatory response of HCC (24–26). Moreover, many dysregulated miRNAs have been demonstrated to act as diagnostic and prognostic factors for HCC patients (27, 28). However, the dysregulation mechanism of miRNAs in the development and progression of HCC remains unclear.

In this work, we found 114 disordered mature miRNAs in HCC, 93 and 10 of which are caused by dysregulated transcription factors and the DNA methylation of promoter region, respectively. We identified 14 disordered miRNAs as prognostic factors for HCC patients, seven up-regulated miRNAs can serve as oncogenes to promote HCC tumorigenesis, and other seven down-regulation miRNAs can act as tumor suppressors to suppress abnormal cell proliferation. Among 14 above prognostic miRNAs, 12 disordered miRNAs were caused by the disorders of 14 transcription factors. Finally, we demonstrated that several up-regulated transcription factors can activate several oncogenic miRNAs to inhibit tumor-suppressing transcription factors FOXO1 to down-regulate a certain number of tumor-suppressing miRNAs, which leaded to the occurrence and development of HCC. Taken together, our results revealed the disordered mechanisms of miRNAs and their prognostic roles in HCC.

## Materials and Methods

### Data Acquisition and Preprocessing

The expression data and clinical information of all HCC samples were generated from the Cancer Genome Atlas (TCGA) (https://portal.gdc.cancer.gov/). These level 3 expression data of mRNA, mature miRNA and pre-miRNA of HCC as well as 12 other tumor types were downloaded from the UCSC Xena (https://xenabrowser.net/). After removing samples with survival days less than 15, we finally retained 344 cancer samples, including 48 matched tumor and paracancerous samples. Next, these samples were randomly divided into independent training sets (240 samples) and test sets (104 samples). The protein-coding gene annotation was derived from the ENSEMBL (http://asia.ensembl.org/index.html), and the human transcription factor annotation was derived from AnimalTFDB 3.0 (http://bioinfo.life.hust.edu.cn/AnimalTFDB/#!/).

### Differentially Expressed Gene Analysis

Differential expression analysis of paired cancer and paracancerous mRNA was performed using the edgeR package (29). Genes with extremely low expression (Sums (cpm) <1) or expressed in no more than half of the sample were removed. The filtering criteria for differentially expressed genes were set to |log2FC| >1, FDR <0.05. The expression level of mRNA was taken log2 logarithm after normalized by edgeR to be used for next analysis. At the same time, differentially expressed miRNAs were identified based on 48 matched cancer and adjacent tissues using the limma package (30). The filtration criteria of differentially expressed miRNA were same as above.

### Methylation-Driven miRNA Analysis

Genome-wide lllumina HumanMethylation450K BeadChip data of HCC samples were derived from UCSC Xena (https://xenabrowser.net/). The methylation value of the methylation site of the sample is represented by the beta value, which was obtained *via* calculating the ratio of the fluorescence signal of the methylation site and the non-methylation site, Beta = M/(M + UM). The H3K4me3 (Accession number: ENCFF219ZOU) and H3K27ac (Accession number: ENCFF259DOA) ChIP-seq data of HCC cell line HepG2 were downloaded from the ENCODE database (https://www.encodeproject.org/). Transcription start sites (TSS) of all human miRNAs were acquired from the mirTrans database (https://mcube.nju.edu.cn/jwang/lab/soft/mirtrans/) (31). The promoter region of miRNA is defined as 1,500 bp upstream and 500 bp downstream of the TSS. These differential methylation sites in matched tumor and adjacent tissues were calculated using Wilcoxon rank sum test. Differential methylation sites were defined as |log2FC| >1 and FDR <0.05. The methylation value is defined as an average value of all methylation sites of miRNA promoter region. The correlation between the methylation value of miRNA promoter region and miRNA expression level was calculated using the spearman method with *p <*0.05.

### Prediction of Transcription Factor Targeting miRNAs

We used this TransmiR2.0 database (http://www.cuilab.cn/transmir) to identify the candidate transcription factor-miRNA regulatory pairs, which were performed by integrating public ChIP-seq, transcription factor binding sites (motif), accurate miRNA transcription start site and manual literature collection (32). Herein, we presumed that transcription factor activates the expression of target miRNA, thus the expression level between transcription factors and miRNAs was positive correlation (*r >*0.2, *p <*0.05), which could be further accepted for a veritable transcription factor-miRNA pair.

### Establishment of miRNA Survival Prognosis Model

The training set was used to screen survival-associated miRNAs and establish prognostic model. The test set was used to verify the reliability and accuracy of the prognostic model. Firstly, the multivariate cox regression analysis was used to screen differentially expressed miRNAs using age and sex as covariates. MiRNAs with *p <*0.05 by the multivariate cox analysis were selected as candidate biomarkers. Then, these candidate miRNAs were divided into high-expression and low-expression groups according to their respectively median expression level to further verify these screened survival-related miRNAs. The multi-gene predictive model was constructed according to these final survival-related miRNAs to predict the risk value of HCC patient, and the survival curve of different risk stratification was plotted to test whether the model could predict the survival of HCC patient. The Receiver Operating Characteristic (ROC) curve was drawn using the survivalROC R package (https://CRAN.R-project.org/package=survivalROC), and the reliability of the prediction model was evaluated based on the AUC curve. In addition, both sensitivity and specificity of prognostic model were verified in the test set.

### miRNA Target Prediction and Target Function Analysis

These target prediction results of miRNAs were derived from miRWalk3.0 (http://mirwalk.umm.uni-heidelberg.de/), which comprehensively contain the miRNA target genes of humans, mice and other species through integrating the TarPmiR algorithm and the information from miRDB, TargetScan, miRTarBase and other databases (33, 34). Since miRNAs negatively regulate their target genes, the spearman correlation coefficient and significance between miRNA and their target genes were calculated. Only those targets with a significant negative correlation with miRNAs (*r* <−0.2, *p <*0.05) were further considered as true targets of miRNAs. Kyoto encyclopedia of Genes and Genomes (KEGG) enrichment analysis of targets was performed using the clusterProfiler package (35).

### Data Statistics and Visualization

All data analyses were performed using the R software version 3.5.1. The network visualization was performed using the cytoscape software 3.6.1. The survival curve was drawn by Kaplan–Meier (K–M), and the difference significance was evaluated by log-rank test.

## Result

### Differentially Expressed miRNAs and Genes

We here identified 114 differentially expressed mature miRNAs including 21 up-regulated and 93 down-regulated mature miRNAs (|logFC| >1, FDR <0.01) (**Figure S1A**), and 1,784 differentially expressed protein coding genes consisting of 1,065 up-regulated and 719 down-regulated genes (|logFC| >1, FDR <0.01) between 48 matched tumor and adjacent tissues (**Figure S1B**). Some 80 up-regulated and 51 down-regulated transcription factors were included in these differentially expressed mRNAs. Remarkably, these abnormally down-regulated miRNAs were nearly 4.3 times higher than these up-regulated miRNAs. Unsupervised clustering heat maps of miRNAs (**Figure S1C**) and mRNAs (**Figure S1D**) further demonstrated significant differences of gene expressions between tumor and adjacent tissues. These above results not only confirmed the reliability of this data source, but indicated a clear grading between tumor and adjacent tissues.

### The Underlying Mechanism Driving miRNA Expression Dysregulation in HCC

**Figure 1:**
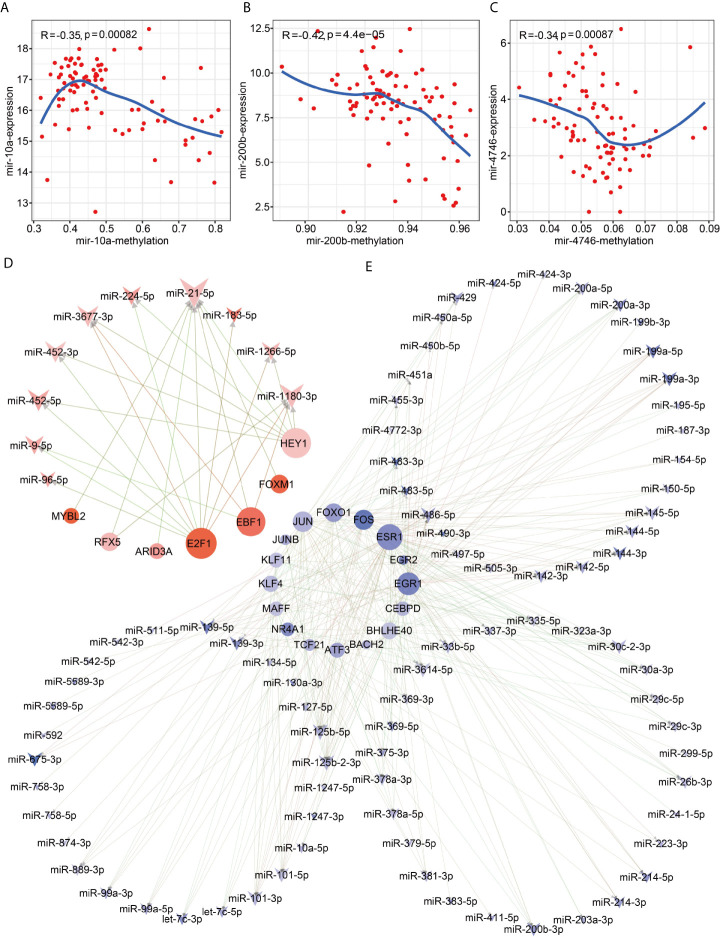
Methylation drivers and transcription factor imbalances cause dysregulated miRNA expression. Correlation between expression and promoter region methylation of pre-miRNAs. **(A)** Spearman correlation diagram of mir-10; **(B)** Spearman correlation diagram of mir-200b; **(C)** Spearman correlation diagram of mir-4746. Network diagram of transcription factors regulating miRNAs. **(D)** Up-regulated transcription factors regulate up-regulated miRNAs; **(E)** Down-regulated transcription factors regulate down-regulated miRNAs.

To further explore the mechanisms of causing disordered expressions of 114 mature miRNAs in HCC, we firstly investigated methylation levels of 114 disordered miRNAs, finding that 10 of them are driven by DNA methylation of their promoter regions (**Table S1**). Especially, the expression levels of miR-10a (*r* <−0.35, *p <*0.001), miR-200b/miR-200a/miR-429 family (*r* <−0.42, *p <*0.001) and miR-4746 (*r* <−0.34, *p <*0.001) are significantly negative correlation with the hypermethylation levels of their promoter regions, respectively (**Figures 1A–C**). These results were consistent with the ChIP-seq data of H3K4Me3 and H3K27ac, which are markers of the active promoter region. Our results showed that the hypermethylated promoter region of miR-323a, miR-376c, miR-154, miR-10a, miR-200a, miR-200b, miR-429 had no active markers in HCC cell line HepG2, but the hypomethylated promoter region of miR-1180 and miR-4746 were obviously enriched in H3K4Me3 and H3K27ac peaks (**Figure S2**). In particular, the promoters of miRNAs belonging to the same family had similar modifications of H3K4Me3 and H3K27ac, such as miR-323a, miR-376c, miR-154 of the miR-323a family and miR-200a, miR-200b, miR-429 of the miR-200 family (**Figure S2**). These results indicated that DNA methylation can drive the expression disorders of 10 mature miRNAs *via* inhibiting the activity of their promoter regions in HCC. Secondly, to detect whether dysregulated transcription factors can result in the disorders of miRNAs in HCC, we predicted these disordered miRNAs regulated by dysregulated transcription factors. Interestingly, we found that 23 dysregulated transcription factors could cause the expression disorders of 93 mature miRNAs (*r >*0.2, *p <*0.05) (**Figures 1D, E** and **Table S2**). Among these regulatory pairs, seven up-regulated transcription factors could activate the up-regulated expressions of 10 mature miRNAs (**Figure 1D**). In contrast, 16 down-regulated transcription factors could down-regulate the expressions of 83 mature miRNAs (**Figure 1E**). Overall, our results suggested that dysregulated transcription factors and DNA methylation are main cause of resulting in the expression disorder of miRNAs in HCC. Moreover, the disordered mechanisms of 11 other miRNAs in HCC have yet not been explored and need further in-depth study.

### Identification of Survival-Related miRNAs and Establishment of Prognostic Model

**Figure 2:**
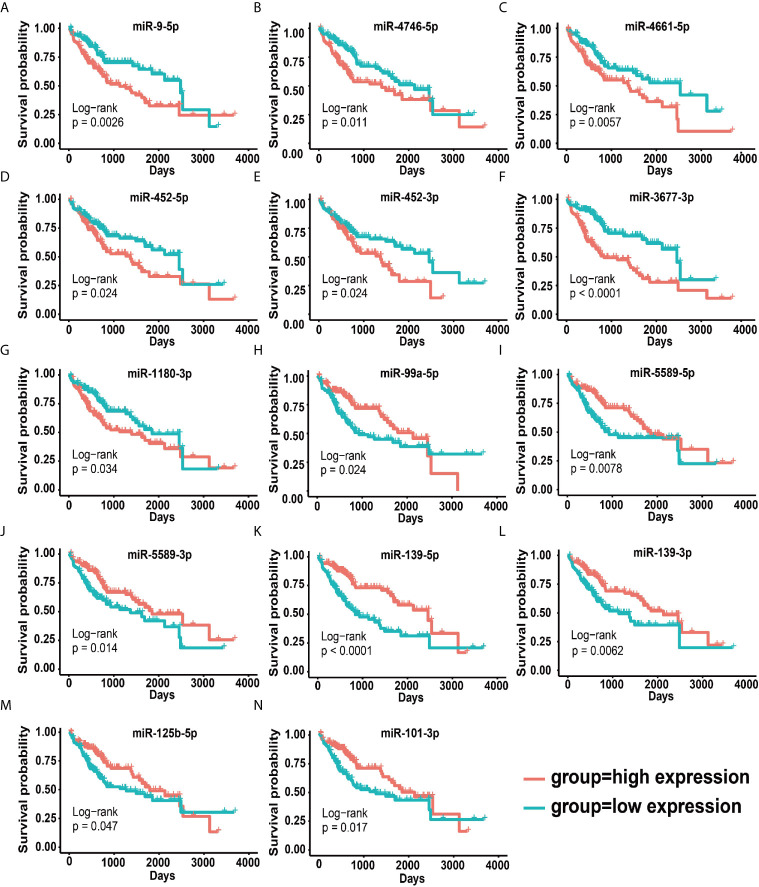
Survival curves for 14 prognostic miRNAs. **(A–H)** Survival curves of seven up-regulated oncogenic miRNAs. **(I–N)** Survival curves of seven down-regulated cancer suppressor miRNAs.

**Figure 3:**
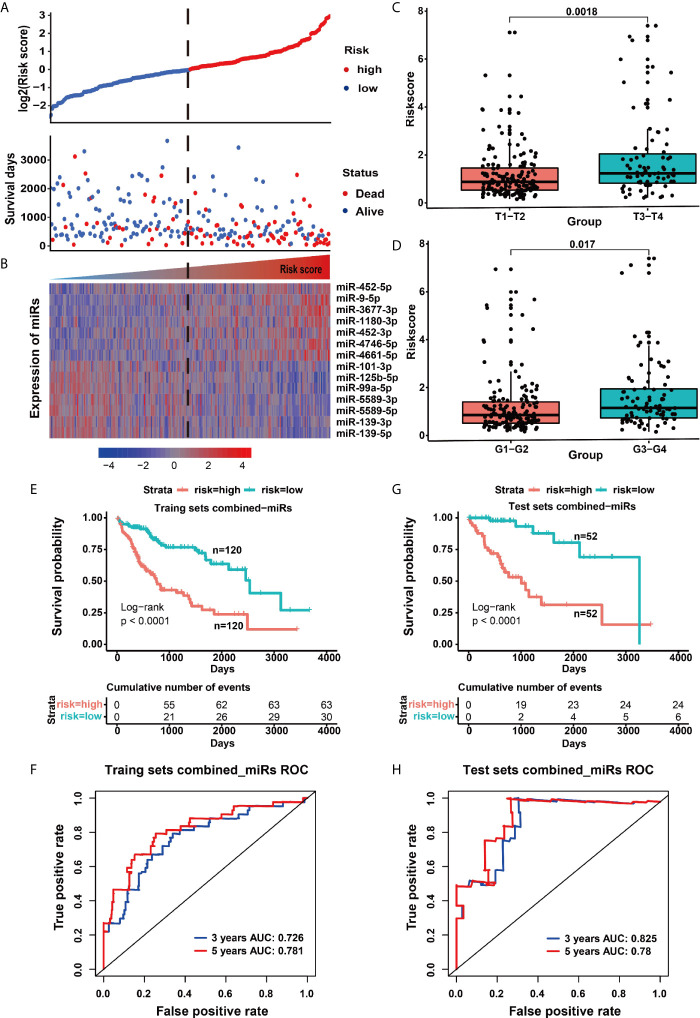
Model risk signature based on 14 miRNAs was used to predict the outcome of patients. **(A)** The distribution map of patient deaths at different risk values. **(B)** The heat map of prognostic miRNA expression in patients with different risk values. **(C)** The risk difference for patients with different T grades (extent of the primary tumor). **(D)** The risk difference for patients with different G grades (histopathological degrees). **(E)** Survival curves of the high and low risk groups in the training set. **(F)** ROC curve of signatures based on 14 miRNAs in the training set. **(G)** Survival curves of the high-risk and low-risk groups in the test set. **(H)** ROC curve of the 14 miRNA-based signatures in the test set.

To reveal whether 114 disordered miRNAs can serve as prognostic and diagnostic factors for HCC patients, we executed the multivariate analysis and the Kaplan–Meier survival analysis for these 114 miRNAs using age and gender as covariates in the training set. We identified 14 prognostic-related miRNAs, including seven down-regulated miRNAs (miR-99-5p, miR-5589-5p, miR-5589-3p, miR-139-5p, miR-139-3p, miR-101-3p and miR-125b-5p) (**Figure S3A**) and seven up-regulated miRNAs (miR-9-5p, miR-452-5p, miR-452-3p, miR-1180-3p, miR-4746-5p, miR-3677-3 and miR-4661-5p) in tumor tissues (**Figure S3B**). Our results revealed that seven up-regulated miRNAs were closely related to the poor prognosis of HCC patients (*p <*0.05) (**Figures 2A–G** and **Figure S3C**), but the other seven down-regulated miRNAs may act as protective miRNAs to promote the good prognosis of HCC patients (*p <*0.05) (**Figures 2H–N** and **Figure S3C**). Furthermore, our results demonstrated that along with the risk increasing, the number of dead patients became more and more denser, and the expression levels of the risk factor miRNAs were gradually increased (**Figure 3A**), whereas the expression levels of the protective factor miRNAs were gradually decreased (**Figure 3B**). Remarkably, patients with high tumor progression (T3–T4 and G3–G4) had significantly higher risk values than patients with low tumor progression (T1–T2 and G1–G2) (*p <*0.05) (**Figures 3C, D**). Especially, the median survival rate of HCC patients in the high-risk group was significantly lower than that in the low-risk group (*p <*0.0001), and the cumulative number of deaths in the high-risk group was also about two times higher than the low-risk group at each cut-off time point (**Figure 3E**). Moreover, the ROC curve based on the 14 miRNAs as a signature demonstrated an average 3 and 5 year AUC values for 0.726 and 0.781, respectively (**Figure 3F**).

To confirm the accuracy of the above prognostic model, we further verified the reliability of the model in the test set. Similar to the training set, the survival rate of HCC patients in the high-risk group was also lower than that in the low-risk group (*p <*0.0001), and the cumulative number of deaths in the high-risk group was more four times than that of the low-risk group (**Figure 3G**), as well as the ROC curve showed a good specificity and sensitivity with 3-year AUC for 0.825 (**Figure 3H**). These above results indicated that the predictive model had a good specificity and sensitivity. Moreover, we further used the multivariate cox analysis to reveal the effect of different clinical factors on the survival of HCC patients. When age, gender, American Joint Committee on Cancer (AJCC) stage and the signature of combined 14 miRNAs were taken as continuous variables for multivariate cox analysis, this prognostic signature was still a robust predictor, whether in the training set (HR = 2.683, *p <*0.001) or the test set (HR = 6.388, *p <*0.001) (**Table 1**). Taken together, our present study revealed that the 14 miRNAs are closely related to the survival and disease progression of HCC patients, implying that they could serve as important prognostic biomarkers for the survival of HCC patients.

**Table 1:** Multivariate analysis of impact on patient survival of risk signature, age, gender and AJCC stage.

Variables	Train set		Test set	
	Hazard ratio (95%CI)	*P*-value	Hazard ratio (95%CI)	*P*-value
Age	1.158 (0.742–1.806)	0.519	1.358 (0.606–3.041)	0.457
Gender	0.828 (0.531–1.290)	0.404	1.199 (0.432–3.330)	0.728
AJCC stage	2.555 (1.643–3.972)	<0.001	1.898 (0.811–4.443)	0.140
Risk signature	2.683 (1.691–4.257)	<0.001	6.388 (2.391–17.071)	<0.001

High and low risk group, age, gender and AJCC stage are coded as continuous variables. Specifically, high-risk group = 1, low-risk group = 0; male = 1, female = 0; AJCC stage I = 1, AJCC stage II = 2, AJCC stage III = 3, AJCC stage IV = 4.

### The Clinical Diagnostic Value of 14 Prognostic miRNAs

To explore the clinical diagnostic value of 14 prognostic miRNAs, we further plotted their respective ROC curves to evaluate the ability of each miRNA expression level in distinguishing tumor from normal tissues. Herein, the comprehensive diagnostic results of AJCC stage was used as indicators of disease progression of HCC patients. Our results indicated that in early diagnosis (AJCC stage I*), the AUC value of each miRNA reached above 0.63, which was higher than the alpha-fetoprotein (AFP) (the AUC value for 0.606) (**Figure S4**). Especially, miR-139-5p, miR-139-3p and miR-101-5p had more high AUC values for 0.958, 0.991 and 0.950, respectively (**Figure S3**). In advanced diagnosis (AJCC stage III*/IV*), the AUC value of each miRNA reached above 0.68, which was higher than AFP (the AUC value of 0.622) (**Figure S5**). Significantly, miR-139-5p, miR-139-3p, miR-101-5p and miR-125b-5p also had very high AUC values for 0.950, 0.985, 0.933 and 0.912, respectively (**Figure S5**). Overall, these above results suggested that these 14 miRNAs may be applied to the diagnosis and detection of HCC patients.

Herein, we further investigated whether these 14 prognostic miRNAs possess specificity or universality. We thus calculated their expressions in other 12 kinds of tumors, finding that the expression levels of miR-4661-5p and miR-452-5p were significantly increased only in one or two kinds of tumors, while miR-5589-3p and miR-5589-5p were also significantly down-regulated just in one tumor (**Figure S6**), which implied that miR-4661-5p, miR-452-5p, miR-5589-3p and miR-5589-5p had higher specificity than other miRNAs. In contrast, miR-4746-5p, miR-3677-3p and miR-1180-3p were respectively significantly up-regulated in five or six types of tumors, meanwhile miR-139-3p, miR-139-5p and miR-99a-5p were also respectively significantly down-regulated in more than six types of tumors (**Figure S6**), implying that their expressions have obvious universality. Additionally, miR-9-5p and miR-452-3p were respectively significant up-regulated in three or four kinds of tumors, and miR-125b-5p and miR-101-3p were also respectively significant down-regulated in three or four tumor types, revealing their moderate specificity (**Figure S6**). These results reveald that these 14 prognostic markers for HCC were both specific and universal, in particular miR-4661-5p, miR-452-5p, miR-5589-3p and miR-5589-5p may be more suitable for the diagnosis and prognosis of HCC patients, as well as the other miRNAs may be also applied to the prognostic and detection markers for other types of tumors.

### Functional Roles of 14 Prognostic miRNAs in HCC

**Figure 4:**
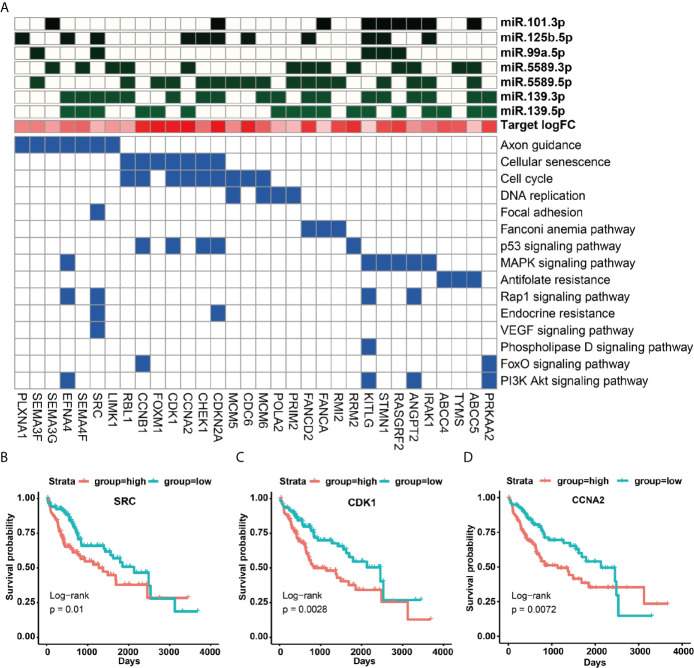
KEGG signaling pathways of up-regulated target genes and their regulation by miRNAs. **(A)** KEGG signaling pathways involved in survival-related up-regulated target genes and their regulation by miRNAs. **(B–D)** The impact of up-regulated target genes on the overall survival rate of HCC patients, taking SRC, CDK1 and CCNA2 as examples.

To understand the function roles of the 14 prognostic miRNAs in the occurrence process of HCC, we predicted their target genes. Our results demonstrated that seven down-regulated miRNAs could target 592 up-regulated genes, and seven up-regulated miRNAs could target 481 down-regulated genes (**Table S3**). To better describe the characteristics of 14 miRNAs and their target genes, we detailedly investigated their target types (carcinogenic, tumor suppressor and cancer-driven genes) recorded by this CancerMine database (http://bionlp.bcgsc.ca/cancermine), and picked out these target genes directly related to the survival of HCC patients to explore they involved in KEGG pathways. Our results indicated that most up-regulated target genes had been identified as carcinogenic genes for the poor prognosis of HCC patients *via* activating cancer-related pathways (**Figure 4A**, **Figure S7A** and **Tables S3**, **S4**). For example, the proto-oncogene *SRC* can involve in Focal adhesion, Rap1, Endocrine resistance, VEGF and other pathways (**Figure 4A** and **Table S4**). Especially, this *SRC* gene can be synergistically inhibited by miR-125b-5p, miR-99a-5p, miR-139-3p, miR-139-5p (**Figure 4A** and **Tables S3**, **S4**), and thus their down-regulation expressions can cause the up-regulation expression of *SRC* to further lead to a poorer prognosis for HCC patients (*p* = 0.01) (**Figure 4B**). Interestingly, studies had reported that miR-99a is induced to be down-regulated when *c-Src* is activated, and re-overexpressed miR-99a can target mTOR/FGFR3 to inhibit Src-related oncogenic pathways and thereby inhibit the growth of lung cancer cells (36). Similarly, down-regulated miR-5589-5p, miR-139-3p, miR-139-5p and miR-125b -5p can synergistically promote the up-regulated expression of oncogenic genes *CDK1* and *CCNA2* to result in the significantly poor prognosis for HCC patients (*p <*0.01) (**Figures 4A, C, D** and **Tables S3**, **S4**). The miR-125b had been reported to inhibit the proliferation of esophageal squamous cell carcinoma cells by reducing *CCND1*, *CCNA2* and *CCNE1* (37). In the mouse model of macrophages, *CCNA2* had also been reported to be targeted by miR-125b (38).

**Figure 5:**
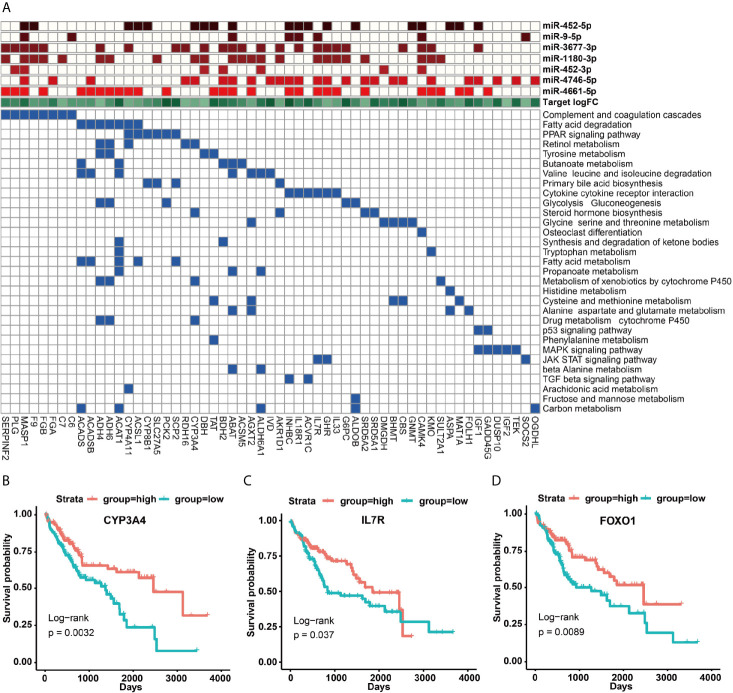
KEGG signaling pathways of down-regulated target genes and their regulation by miRNAs. **(A)** KEGG signaling pathways involved in survival-related down-regulated target genes and their regulation by miRNAs. **(B–D)** The impact of up-regulated target genes on the overall survival rate of HCC patients, taking CYP3A4, IL7R and FOXO1 as examples.

Unlike these up-regulated target genes, these down-regulated target genes of seven up-regulated miRNAs were more enriched in metabolic and immune-related signaling pathways (**Figure S7B**), in particular many down-regulated target genes could be acted as tumor suppressor genes (**Figure 5A** and **Table S5**). For example, the up-regulated expressions of miR-452-5p, miR-1180-3p and miR-4746-5p could significantly inhibit the expression of the tumor suppressor gene *CYP3A4* to cause a remarkably poor prognosis of HCC patients (*p* = 0.0032) (**Figures 5A, B** and **Tables S3**, **S5**). Interestingly, miR-452 had been demonstrated to target *CYP2C8* and the same cluster of miR-224 can target *CYP3A4* to affect metabolism and detoxification in the liver (39). Besides, *IL7R* was involved in JAK-STAT and cytokine interaction pathway, and the up-regulated expression of miR-9-5p repressed *IL7R* expression to further lead to a poorer prognosis of HCC patients (*p* = 0.037) (**Figures 5A, C**). Moreover, IL7R and other interleukin genes have been reported to be inhibited by miR-9, which may link inflammation with nasopharyngeal carcinoma (40).

### Roles of Transcription Factors in HCC Occurrence

Herein, we found 14 transcription factors could regulate 12 prognostic miRNAs, i.e. four up-regulated transcription factors (E2F1, EBF1, HEY1 and RFX5) could activate the expressions of miR-9-5p, miR-452-5p, miR-452-3p, miR-3677-3p and miR-1180-3p, as well as 10 down-regulated transcription factors (FOXO1, ESR1, FOS, JUN, EGR1, KLF11, MAFF, ATF3, BHLHE40 and CEBPD) could down-regulate the expressions of miR-99a-5p, miR-101-3p, miR-139-5p, miR-139-3p, miR-125b-5p, miR-5589-5p and miR-5589-3p (**Figures 1D, E**). More importantly, our results demonstrated that among 14 prognostic miRNAs, 12 dysregulated miRNAs were caused by the disorders of 14 transcription factors, and one miRNA (miR-4746) was driven by DNA methylation (**Figure 1C**), as well as the abnormal expression mechanism of another miRNA (miR-4661-5p) was still unknown to data. Our results suggested that the dysregulated transcription factors can play vital roles in HCC.

**Figure 6:**
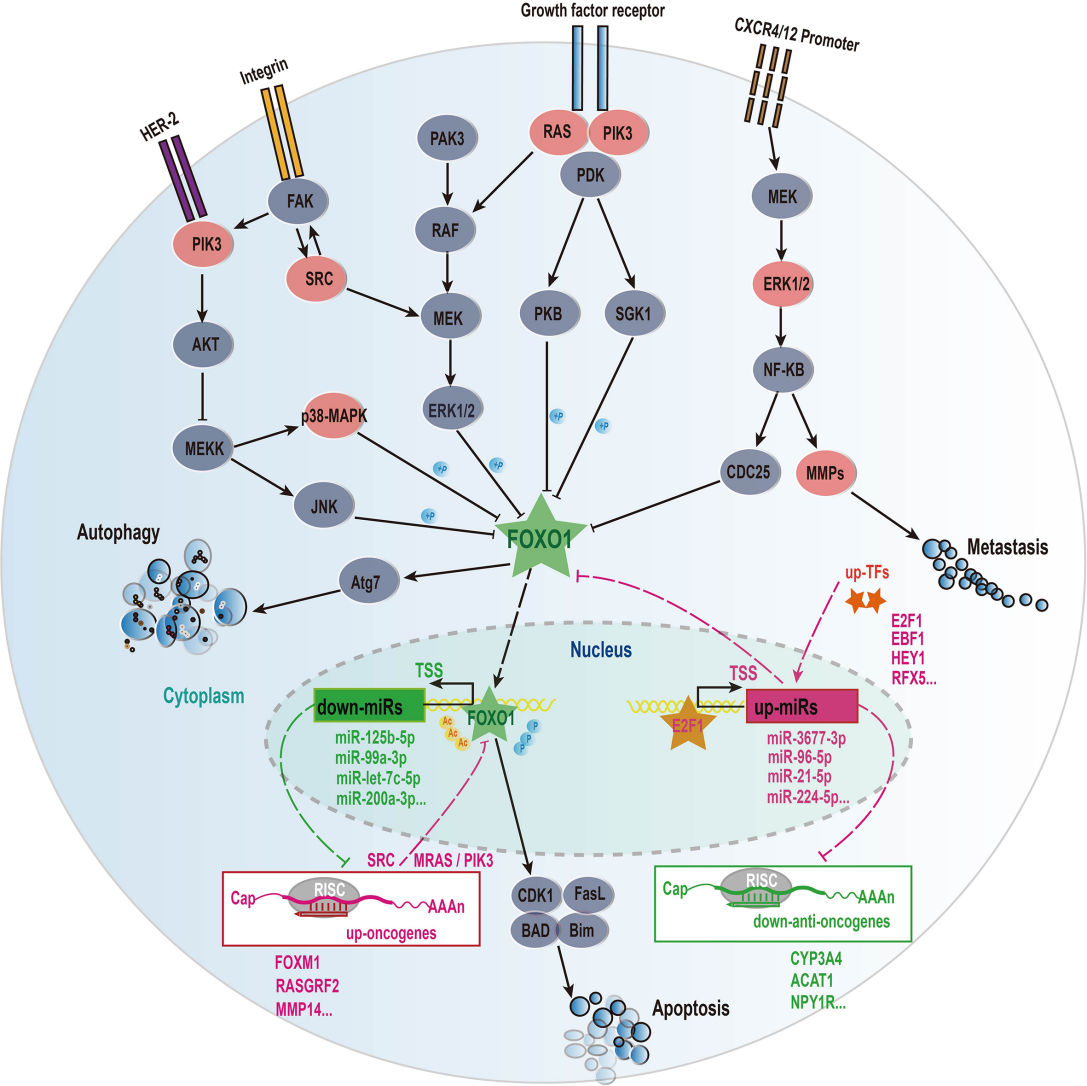
The mechanism of transcription factors and miRNAs interaction affecting the progression of HCC disease. These solid lines represent regulatory relationships and signal pathways that have been experimentally verified, and the dotted lines represent our results of bioinformatics analysis. The molecules involved in the signaling pathway refer to the gene family such as PIK3 family, RAS family, but not the specific gene name. Sharp arrows represent activation and flat arrows represent inhibition. Red represents up-regulated genes and green represents down-regulated genes the difference analysis.

In this work, we further focused on the roles of transcription factor FOXO1 in HCC. Our results indicated that HCC patients in the highly expressed *FOXO1* group exhibited a better survival rate than the lowly expressed *FOXO1* group (*p* = 0.0089) (**Figure 5D**), revealing that *FOXO1* could be served as a tumor suppressor gene to improve the good prognosis of HCC patients, which is agree with these previous studies (41). As shown in **Figure 6**, FOXO1 can enter the nucleus to activate the expression of its target genes to maintain normal cell apoptosis and autophagy in normal cells (41, 42). When cells became cancerous, the upstream cellular pathways of FOXO1 such as PIK3, ERK, RAS, NF-kappa B can phosphorylate or acetylate FOXO1 to cause abnormal cell proliferation and migration to form tumors (41, 42). After phosphorylation, the stability of FOXO1 protein decreased and further leaded to the ability loss of entering the nucleus and shifting from the nucleus to the cytoplasm, thereby losing its ability to regulate downstream target genes (41, 42). Especially, acetyl modification usually affects the ability of FOXO1 to bind to the promoter of its target genes (38, 39). Our results demonstrated that on the one hand the down-regulated *FOXO1* could cause the down-regulation of certain tumor suppressed miRNAs (e.g. miR-125b-5p, miR-99a-5p, miR-101-3p, miR-let-7c, miR-200a-3p) to promote the expressions of upstream carcinogenic genes (e.g. *PIK3C2B*, *PIK3R3*, *SRC*, *EFNA4*, *MRAS*, *RASL1*, *RASGRF2*, *MAPK11*, *MAPK12*, *MMP14*) to activate multiple cancer-related pathways, which further lead to the occurrence of HCC (**Figures 6**, **S8** and **Table S3**); on the other hand several up-regulated transcription factors (e.g. *E2F1*, *EBF1*, *FOXM1* and *RFX5*) might inhibit the expressions of *FOXO1* and certain tumor suppressor genes (e.g. *CYP3A4*, *ACAT1 and NPY1R*) to promote the occurrence of HCC *via* activating carcinogenic miRNAs (e.g. miR-224-5p, miR-96-5p, miR-3677-3p and miR-21-5p) (**Figures 5**, **6** and **Tables S3**, **S5**). These results seemed to imply that transcription factors and miRNAs interplay could suppress the expression of *FOXO1* to participate in the occurrence and development of HCC (**Figure 6**). Overall, our study revealed that FOXO1 dysregulation might cause the dysregulation of a certain number of tumor suppressor miRNAs to result in the occurrence of HCC (**Figures 6** and **S8**), in particular the interplay between transcription factors and miRNAs could synergistically control the occurrence and development of HCC (**Figure 6**).

## Discussion

Although studies have demonstrated that the dysregulated expressions of miRNAs participate in the pathogenesis of HCC (4, 6), the dysregulated mechanisms of causing miRNA in HCC are still poorly understood up to now. In this work, we further study this issue. Our results reveal that transcription factor dysregulations and DNA methylation were two main mechanisms of causing the expression disorders of miRNAs in HCC. Especially, the transcription factor FOXO1 can interplay with multiple prognostic miRNAs forming distinct regulatory loops to involve in the occurrence and development of HCC (**Figure 6**). On the one hand, FOXO1 can be served as a tumor suppressor to activate several protective miRNAs (e.g. miR-125b-5p and miR-99a-5p, miR-101-3p) to further inhibit its upstream carcinogenic genes (e.g. PIK3C2B, PIK3R3, SRC, EFNA4, MRAS, RASL1, RASGRF2, MAPK11, MAPK12, MMP14) of FOXO1 to promote a good prognosis for HCC patients (**Figures 6**, **S8** and **Tables S3**, **S4**). On the other hand, some up-regulated transcription factors (e.g. E2F1 and FOXM1) can activate multiple carcinogenic miRNAs (e.g. miR-21-5p and miR-96-5p) to inhibit FOXO1 to lead to the disorder of FOXO1-miRNA-PI3K/RAS/ERK cancer suppressive feedback loop to cause the occurrence and development of HCC (**Figure 6**). In this work, we found that the down-regulated FOXO1 could control 26 down-regulated miRNAs (**Figure S8**), and at least 10 of them have been reported to act as tumor suppressors in HCC or other cancers (4, 6, 43–50). While we found that four up-regulated miRNAs (miR-224-5p, miR-96-5p, miR-21-5p and miR-3677-3p) might inhibit the expression of FOXO1 (**Figure S8**), of which miR-21-5p and miR-96-5p had also been confirmed to inhibit FOXO1 (51, 52). In addition, our study indicated that several up-regulated transcription factors (e.g. E2F1, EBF1 and FOXM1) might inhibit the expression of FOXO1 *via* activating miR-224-5p, miR-96-5p and miR-21-5p (**Figure 6** and **Table S3**). Interestingly, previous studies had reported that E2F1 can activate the expression of mir-224 and mir-452 to inhibit the tumor suppressor gene *TXNIP* to promote glioblastoma metastasis (53), and that E2F1 and FOXM1 can activate the miR-21-5p and miR-96-5p to inhibit FOXO1 (54–56), which further supports our conclusions.

In this study, we demonstrated that seven up-regulated miRNAs could act as oncogenes (**Figures 2H–N** and **S2C**). Previous studies have revealed that the up-regulated miR-452-3p can promote the proliferation and migration of hepatocellular carcinoma cells *via* targeting *CPEB3/EGFR* axis in HCC (57), which is agreement with our target prediction results (**Table S3**), and this up-regulated miR-1180-3p can involve in the regulation of apoptosis *via* targeting *NF-KB* in HCC (58), as well as miR-3677-3p was significantly up-regulated in cirrhotic patients with antral vasodilation (59). These previous studies further support that miR-452-3p, miR-1180-3p and miR-3677-3p can serve as prognostic miRNAs for HCC patients. Although the roles of other four miRNAs in HCC are yet not reported to date, several other studies have demonstrated that miR-9-5p could increase cancer cells stemness to enhance their resistance to therapy in breast cancer (60), and could promote the proliferation and metastasis of cancer cells in non-small cell lung cancer (61), as well as the highly expressed miR-452-5p was associated with a poor prognosis of patients with renal cell carcinoma (62). Especially, miR-4746-5p was a newly identified prognostic miRNA in our work, which was also found to be significantly up-regulated in more than eight cancer types (63). These above results seemed to imply that miR-9-5p, miR-452-5p and mir-4746-5p can act as new prognostic biomarkers for HCC patients. However, the relationship between miR-4661 and tumors has rarely been reported, which needs further study.

In contrast, the other 7 down-regulated miRNAs could serve as tumor suppressors (**Figures 2A–G** and **S2C**). Especially, miR-99a had been confirmed to be significantly down-regulated in HCC and be appraised as an independent prognostic factor for inhibiting the growth of HCC by inducing cell cycle arrest (64), as well as miR-139-5p as a tumor suppressor gene could target ETS1, *VEGFR* and *SPOCK1* to inhibit cell proliferation and invasion in HCC (19, 65, 66). Moreover, the down-regulated miR-125b-5p could induce the up-regulation of its target *CD16* to promote tumor progression and lead to a poorly clinical prognosis for HCC patients (67), as well as miR-101-3p had been proved to be down-regulated and could target *SOX9* to inhibit cell proliferation and metastasis in HCC (68, 69). Although the roles of miR-139-3p in HCC are yet not reported, it could act as a tumor suppressor gene to involve in HPV-16-induced head and neck cancer, in particular its high expression could reduce the expression of HPV-16 oncogene (70). Considering that viruses could be served as independent risk factors for HCC, and thus we suggest that miR-139-3p might have the similar regulatory mechanism in HCC. Remarkably, miR-5589-5p and miR-5589-3p, as newly identified miRNAs, are still rarely studied and are expected to become new prognostic markers for liver cancer.

At present, targeted miRNA therapy is mainly divided into miRNA silencing and anti-miRNA also called miRNA recovery. Interestingly, silencing several oncogenic miRNAs (e.g. miR-221) by specific antigomirs or anti-miRNA oligonucleotides can play a very good antitumor activity in the prostate (71), and that the oligonucleotides against the miR-221 mouse liver cancer model has demonstrated that its silencing can significantly inhibit tumor cell proliferation and increase apoptosis (72). In addition to the silencing miRNAs, enhancing the expression of tumor suppressor miRNAs is also an effective treatment for HCC patients. For example, increasing miR-26a expression with adeno-associated virus (AAV) can significantly suppress the proliferation of cancer cells, and induce tumor cell apoptosis, as well as protect the treatment group from tumors without side effects (73). The restored expression of miR-375 can also play a good therapeutic effect in a mouse xenograft model (74). Especially, as the first clinical miRNA mimic for HCC, restoring miR-34 expression can inhibit the expression of at least 24 known oncogenes (75). These studies have suggested that targeted miRNA therapy might be an effective treatment for HCC patients.

It is difficult to rebuild the post-transcriptional homeostatic system by normalizing individual miRNA. However, in our study, we found that most dysregulation expression miRNAs can be caused by 23 disordered transcription factors (**Figures 1D, E**). This seems to provide a new opportunity for this awkward situation, i.e. it is conceivable to recover the expression levels of dysfunctional miRNAs *via* restoring the expression levels of several dysregulated transcription factors in HCC, which has at least the following two advantages. First, restoring the normal expression of transcription factors not only can solve its own imbalance, but can control the expression of multiple oncogenic and/or tumor suppressor miRNAs to maintain a new homeostasis. Second, compared with drugs that target miRNAs, the current drug design of proteins is more mature and complete, and people can also make full use of many existing resources. Therefore, we here suggested that targeted transcription factor therapy should be an effective treatment for HCC patients. Interestingly, our study had revealed that this transcription factor FOXO1 can activate 26 disordered tumor suppressor miRNAs to inhibit most carcinogenic genes to promote the survival of HCC patients. This means that the recovery of FOXO1 expression may be a good idea for reconstructing the post-transcriptional regulation of miRNAs to suppress tumorigenesis. A previous study had demonstrated that the overexpression of FOXO1 can significantly increase the expression levels of many target miRNAs (e.g. miR-125b, miR-99, miR-101, miR-let-7c, miR-675, miR-199a) to suppress nasopharyngeal carcinoma cell proliferation (76). Taken together, our study suggested that FOXO1 should serve as an effective therapeutic target for HCC patients.

Here, we also realize that this study has certain limitations. We identify transcription factors regulating miRNAs based on direct activation effects, which may have a certain deviation because some transcription factors may exert inhibitory functions. Therefore, we also predicted the inhibitory effect of transcription factors on miRNAs (**Figure S9**). We found that some down-regulated transcription factors (e.g. JUN and JUNB) may lead to the up-regulation of miR-224-5p, miR-3677-3p and miR-182-5p, and other up-regulated transcription factors can also bind multiple promoter region of negatively correlated miRNAs, but these prediction results need to be carefully verified. Compared with these activated transcription factors, there are yet very few reports on transcription factors directly inhibiting miRNAs to date. In particular, genome mutations, copy number variations, histone modifications, RNA modifications and changes in key proteins, such as DROSHA, DGCR8, XPO5 and AGO2, may also affect miRNA expression (77–80). Therefore, these factors may also drive miRNA dysregulation, which need further study.

## Conclusion

In conclusion, our study not only identified novel prognostic factors and revealed functional roles of prognostic miRNAs in HCC, but systematically illuminated the dysregulated mechanisms of miRNA in HCC. And thus our findings have provided several new and accurate biomarkers for the diagnosis and detection of HCC patients, as well as new insights and methods for targeted therapy for HCC patients.

## Data Availability Statement

The original contributions presented in the study are included in the article/**Supplementary Material**. Further inquiries can be directed to the corresponding authors.

## Author Contributions

SQ and FM designed this study. FM and XX edited this manuscript. SQ acquired and analyzed the data. SQ wrote the original draft. PJ supervised this project. SQ, JX, YY, and SJ collected and investigated literatures. JX, YY, and SJ participated in the commentary of the manuscript. All authors contributed to the article and approved the submitted version.

## Funding

This research was funded by grants from the Natural Science Foundation of Jiangsu Province (No. BK20191368), Key Research & Development Program of Jiangsu Province (No. BE2018713), Jiangsu Provincial Association for Maternal and Child Health Studies Commissioned Research Project Funding (No. JSFY202005), Open subject of Jiangsu Population Society (Nos. JSPA2019017 and JSPA2019020), National Population Commission Open subject (No. YJJC201802) and Priority Academic Program Development of Jiangsu Higher Education Institutions.

## Conflict of Interest

The authors declare that the research was conducted in the absence of any commercial or financial relationships that could be construed as a potential conflict of interest.

## Supplementary Material

Supplementary Figure 1 Volcano and heat maps of differentially expressed genes. **(A, B)** Volcano plots of differentially expressed miRNAs and mRNAs. **(C, D)** Unsupervised clustering heat map of differentially expressed miRNA and mRNA of matched tumor and adjacent tissues. Rows represent genes, columns represent samples, blue represents low expression and red represents high expression in tumor tissue.

Supplementary Figure 2 Use H3K4me3 and H3K27ac ChIP-seq data to verify the promoter activity of 10 miRNAs driven by methylation. **(A)** The chromatin modification of H3K4me3 and H3K27ac in the promoter regions of miR-323a, miR-376c and miR-154. **(B)** Chromatin modification of H3K4me3 and H3K27ac in the promoter region of miR-1180. **(C)** Chromatin modification of H3K4me3 and H3K27ac in the promoter region of miR-4746. **(D)** Chromatin modification of H3K4me3 and H3K27ac in the promoter region of miR-10a. **(E)** Chromatin modification of H3K4me3 and H3K27ac in the promoter regions of miR-200b, miR-200a and miR-429. The red arrow represents the direction of transcription, and the transcription start sites (TSSs) of the prognostic miRNAs refer to **Table S1**. The gray shading represents the promoter region.

Supplementary Figure 3 14 miRNAs related to HCC patient survival. **(A)** Seven up-regulated miRNA expressions in matched tumors and normal tissues. **(B)** Expressions of seven down-regulated miRNAs in paired tumors and normal tissues. **(C)** Forest value map of the risk of 14 prognostic miRNAs affecting the survival time of HCC patients. A HR greater than 1 means that this increasing miRNA expression is not conducive to patient survival, while a HR less than 1 means that this increasing miRNA expression is conducive to patient survival. The absolute value of HR represents the intensity of the effect of miRNA on the survival time of patients.

Supplementary Figure 4 ROC curves of 14 prognostic miRNAs and AFP distinguish early tumor tissue from normal tissue.

Supplementary Figure 5 ROC curves of 14 prognostic miRNAs and AFP distinguish advanced tumor tissue from normal tissue.

Supplementary Figure 6 The expression changes of 14 independent prognostic miRNAs in 12 other tumors types. Only matched tumor and adjacent tissues are used for calculation. The threshold of differentially expressed genes is |logFC| > 1 and FDR < 0.05. Red represents up-regulation, blue represents down-regulation, and gray represents no difference of gene expression. The full name of tumor types are as follows. BLCA, Bladder Urothelial Carcinoma; BRCA, Breast invasive carcinoma; CHOL, Cholangio carcinoma; ESCA, Esophageal carcinoma; HNSC, Head and Neck squamous cell carcinoma; KICH, Kidney Chromophobe; KIRC, Kidney renal clear cell carcinoma; KIRP, Kidney renal papillary cell carcinoma; LUAD, Lung adenocarcinoma; LUSC, Lung squamous cell carcinoma; PRAD, Prostate adenocarcinoma; THCA, Thyroid carcinoma.

Supplementary Figure 7 KEGG pathway enrichment analysis of 14 prognostic miRNA target genes. **(A)** KEGG pathway enrichment analysis of up-regulated target genes. **(B)** KEGG pathway enrichment analysis of down-regulated target genes.

Supplementary Figure 8 Interaction between FOXO1 and miRNAs. Red represents up-regulation of expression, and green represents down-regulation of expression.

Supplementary Figure 9 Network diagram of predicted transcription factor suppression miRNA. The circles represent transcription factors, and the triangles represent miRNAs. Red represents up-regulation and blue represents down-regulation.
