# Supplementary figures and images for: Transcription Factors and Methylation Drive Prognostic miRNA Dysregulation in Hepatocellular Carcinoma
Source: Front Oncol. 2021 Jul 1;11:691115. doi: 10.3389/fonc.2021.691115 (PMC8297977; doi:10.3389/fonc.2021.691115)

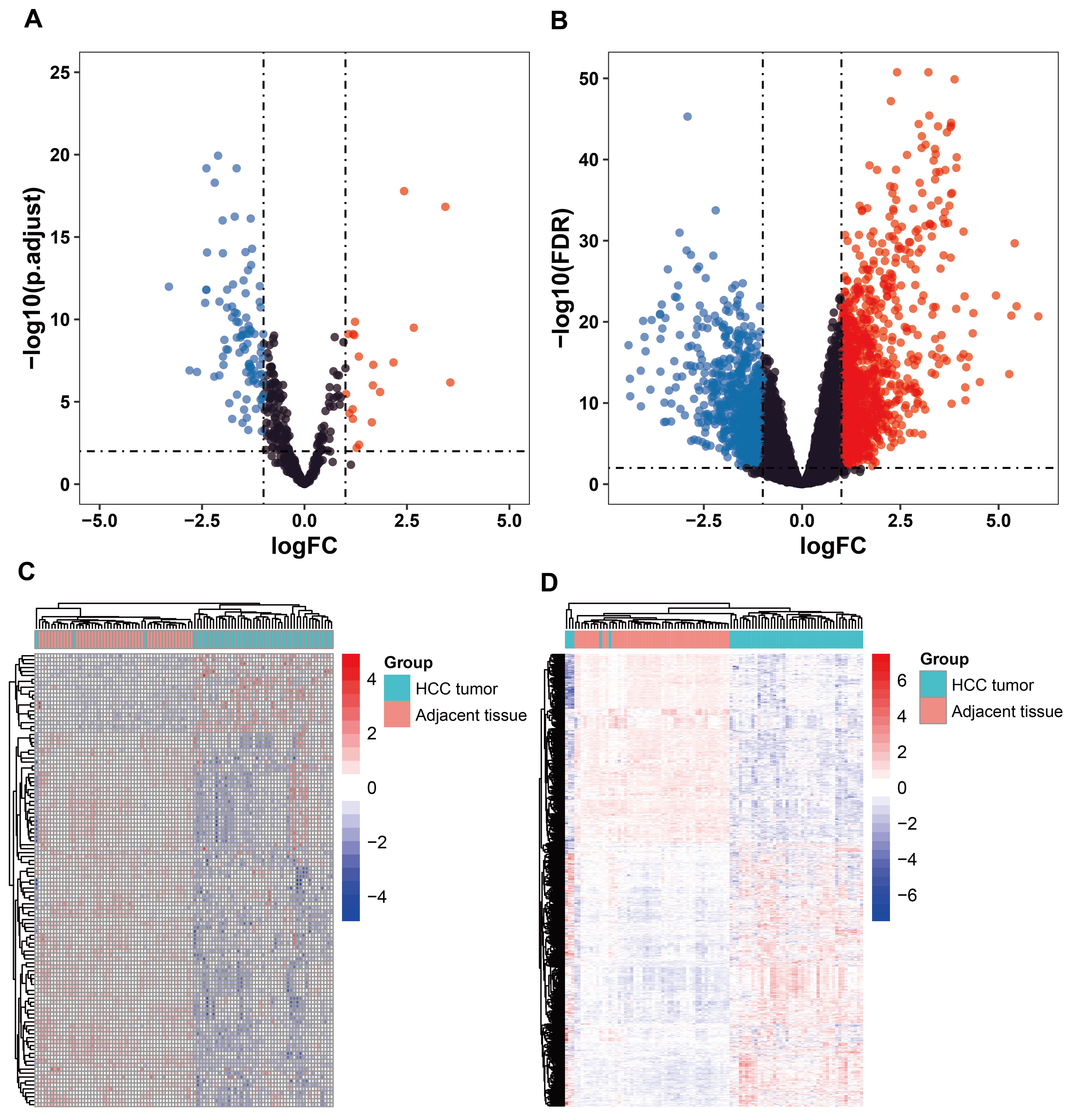

Supplement: Supplementary Figure 1 — Volcano and heat maps of differentially expressed genes. (A, B) Volcano plots of differentially expressed miRNAs and mRNAs. (C, D) Unsupervised clustering heat map of differentially expressed miRNA and mRNA of matched tumor and adjacent tissues. Rows represent genes, columns represent samples, blue represents low expression and red represents high expression in tumor tissue. [file Image_1.jpeg]

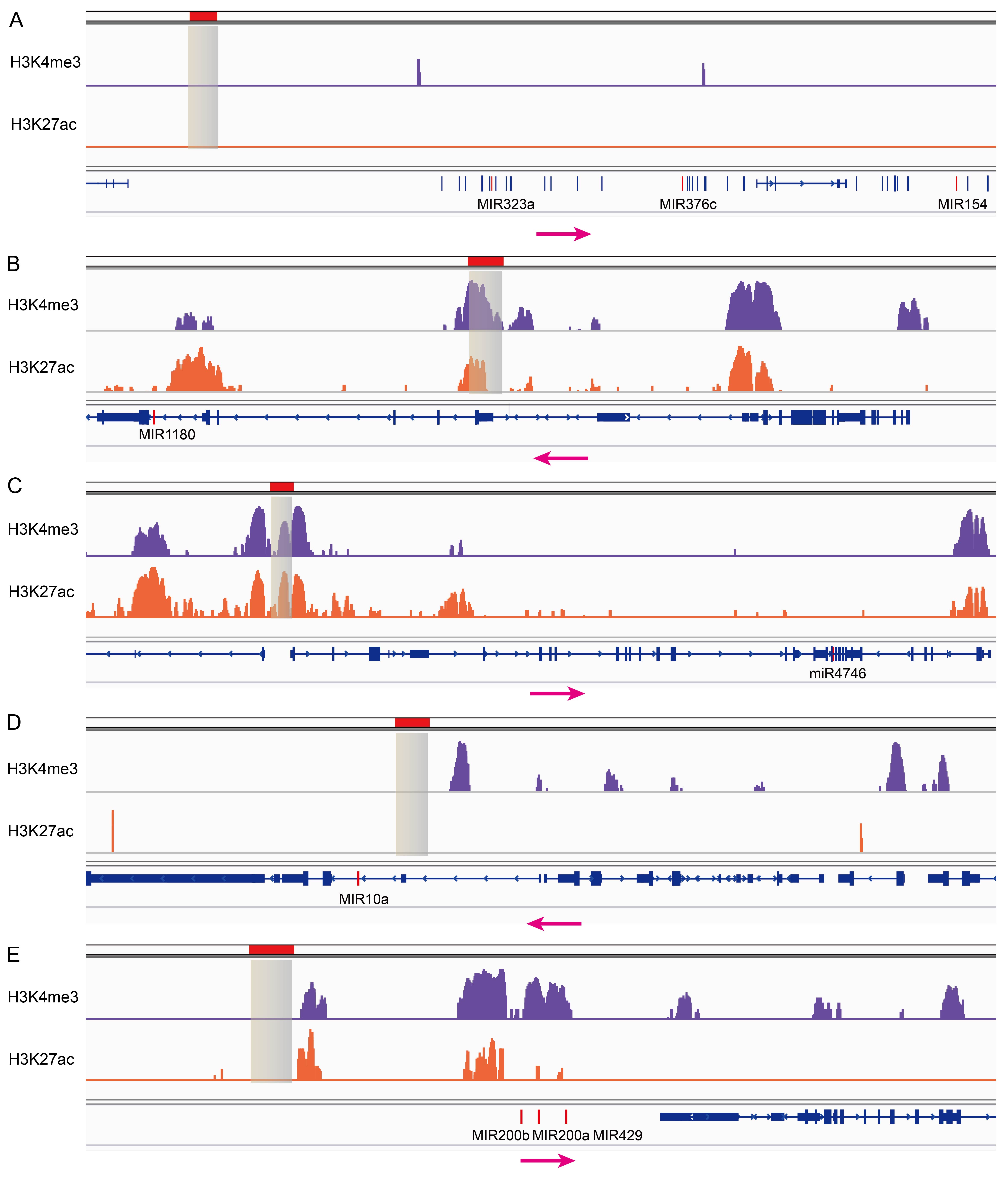

Supplement: Supplementary Figure 2 — Use H3K4me3 and H3K27ac ChIP-seq data to verify the promoter activity of 10 miRNAs driven by methylation. (A) The chromatin modification of H3K4me3 and H3K27ac in the promoter regions of miR-323a, miR-376c and miR-154. (B) Chromatin modification of H3K4me3 and H3K27ac in the promoter region of miR-1180. (C) Chromatin modification of H3K4me3 and H3K27ac in the promoter region of miR-4746. (D) Chromatin modification of H3K4me3 and H3K27ac in the promoter region of miR-10a. (E) Chromatin modification of H3K4me3 and H3K27ac in the promoter regions of miR-200b, miR-200a and miR-429. The red arrow represents the direction of transcription, and the transcription start sites (TSSs) of the prognostic miRNAs refer to Table S1 . The gray shading represents the promoter region. [file Image_2.jpeg]

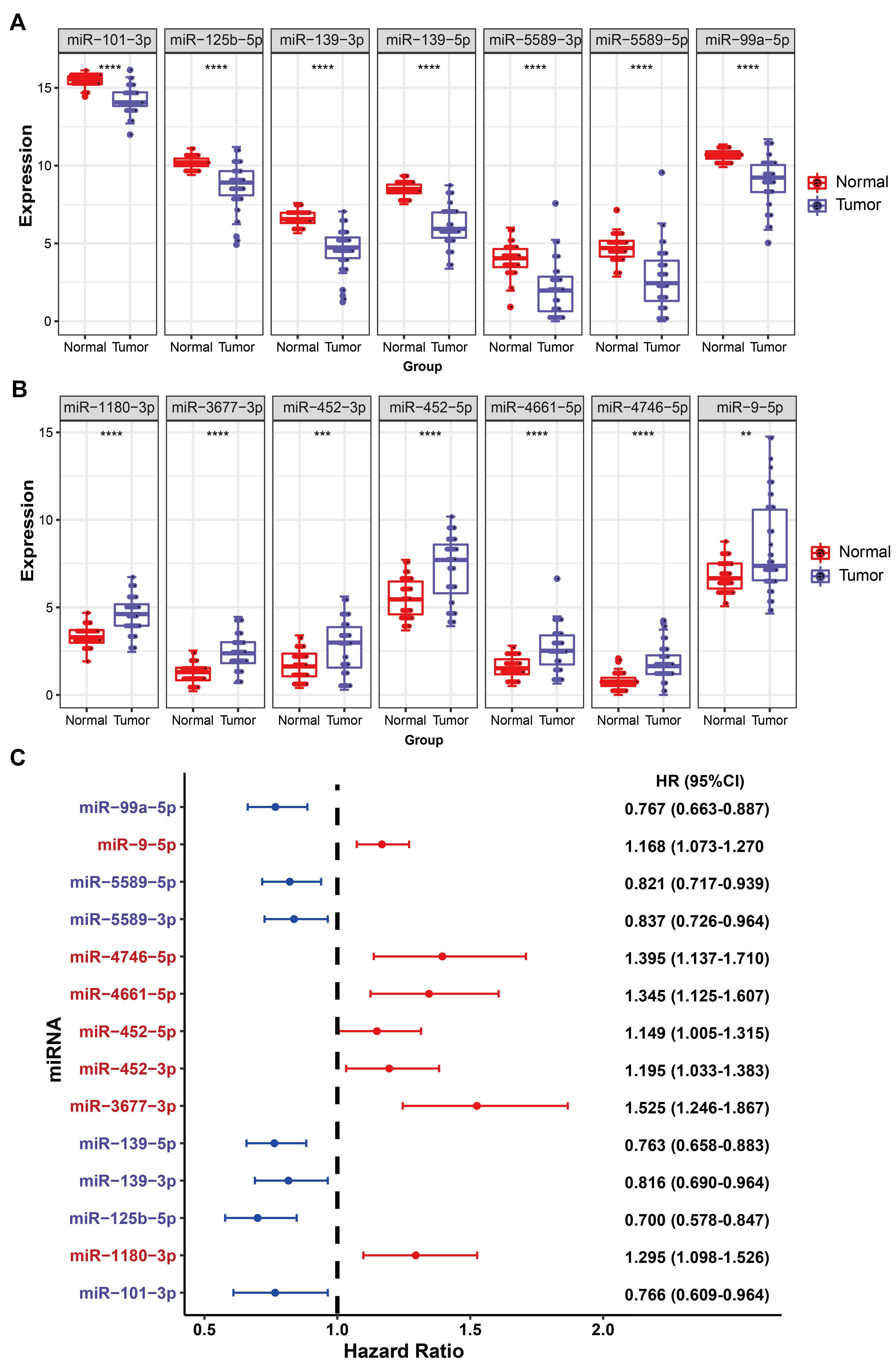

Supplement: Supplementary Figure 3 — 14 miRNAs related to HCC patient survival. (A) Seven up-regulated miRNA expressions in matched tumors and normal tissues. (B) Expressions of seven down-regulated miRNAs in paired tumors and normal tissues. (C) Forest value map of the risk of 14 prognostic miRNAs affecting the survival time of HCC patients. A HR greater than 1 means that this increasing miRNA expression is not conducive to patient survival, while a HR less than 1 means that this increasing miRNA expression is conducive to patient survival. The absolute value of HR represents the intensity of the effect of miRNA on the survival time of patients. [file Image_3.jpeg]

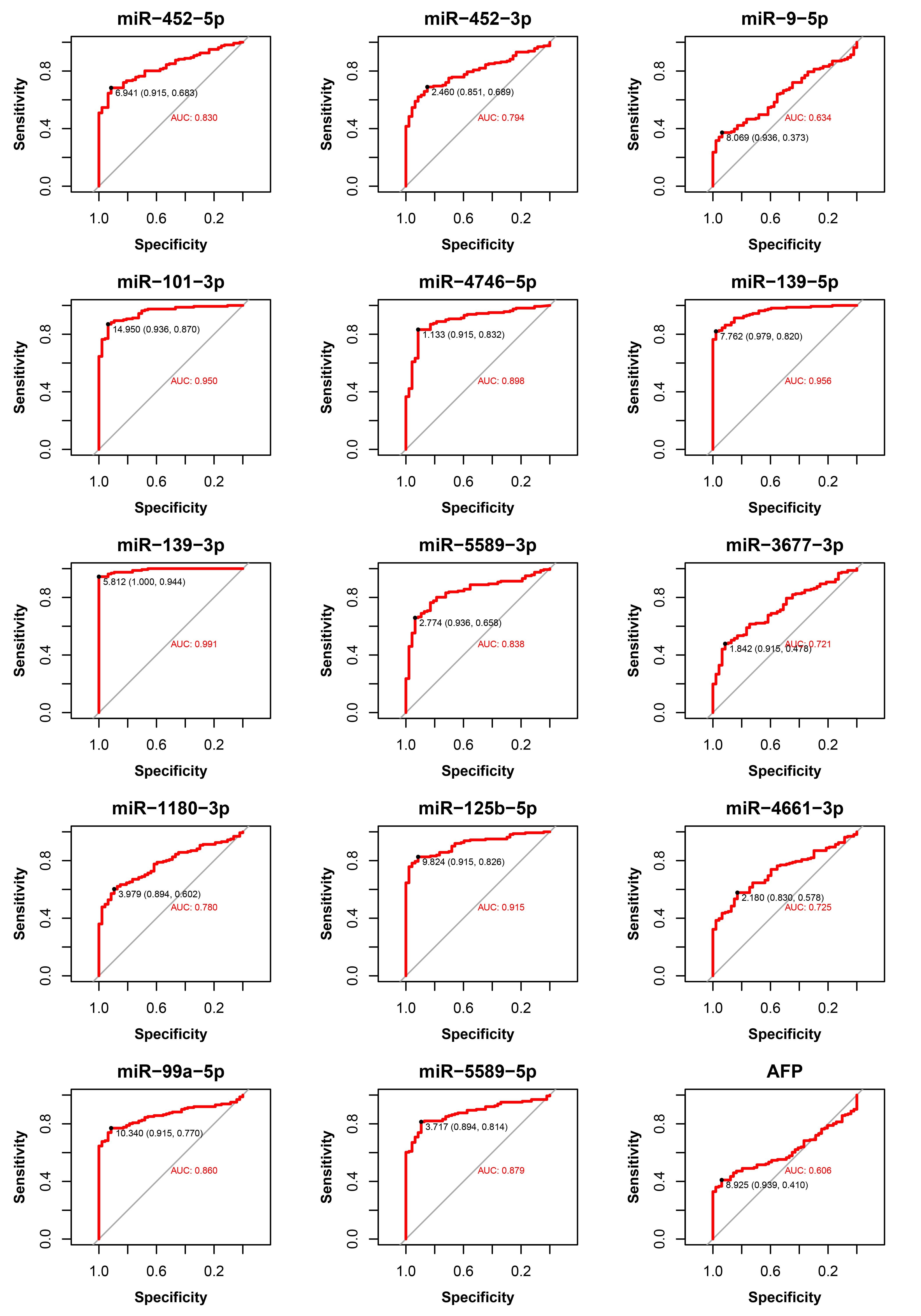

Supplement: Supplementary Figure 4 — ROC curves of 14 prognostic miRNAs and AFP distinguish early tumor tissue from normal tissue. [file Image_4.jpeg]

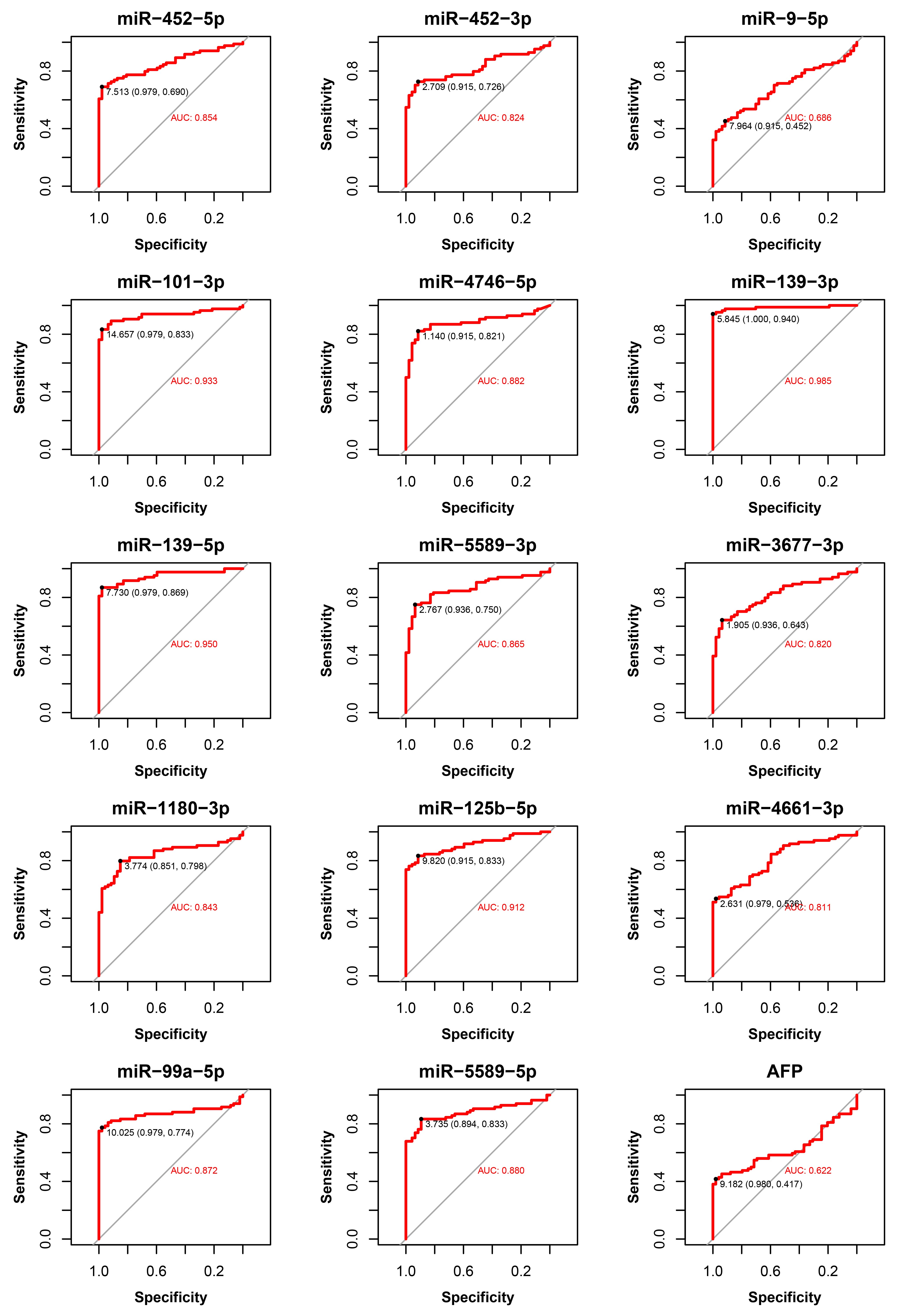

Supplement: Supplementary Figure 5 — ROC curves of 14 prognostic miRNAs and AFP distinguish advanced tumor tissue from normal tissue. [file Image_5.jpeg]

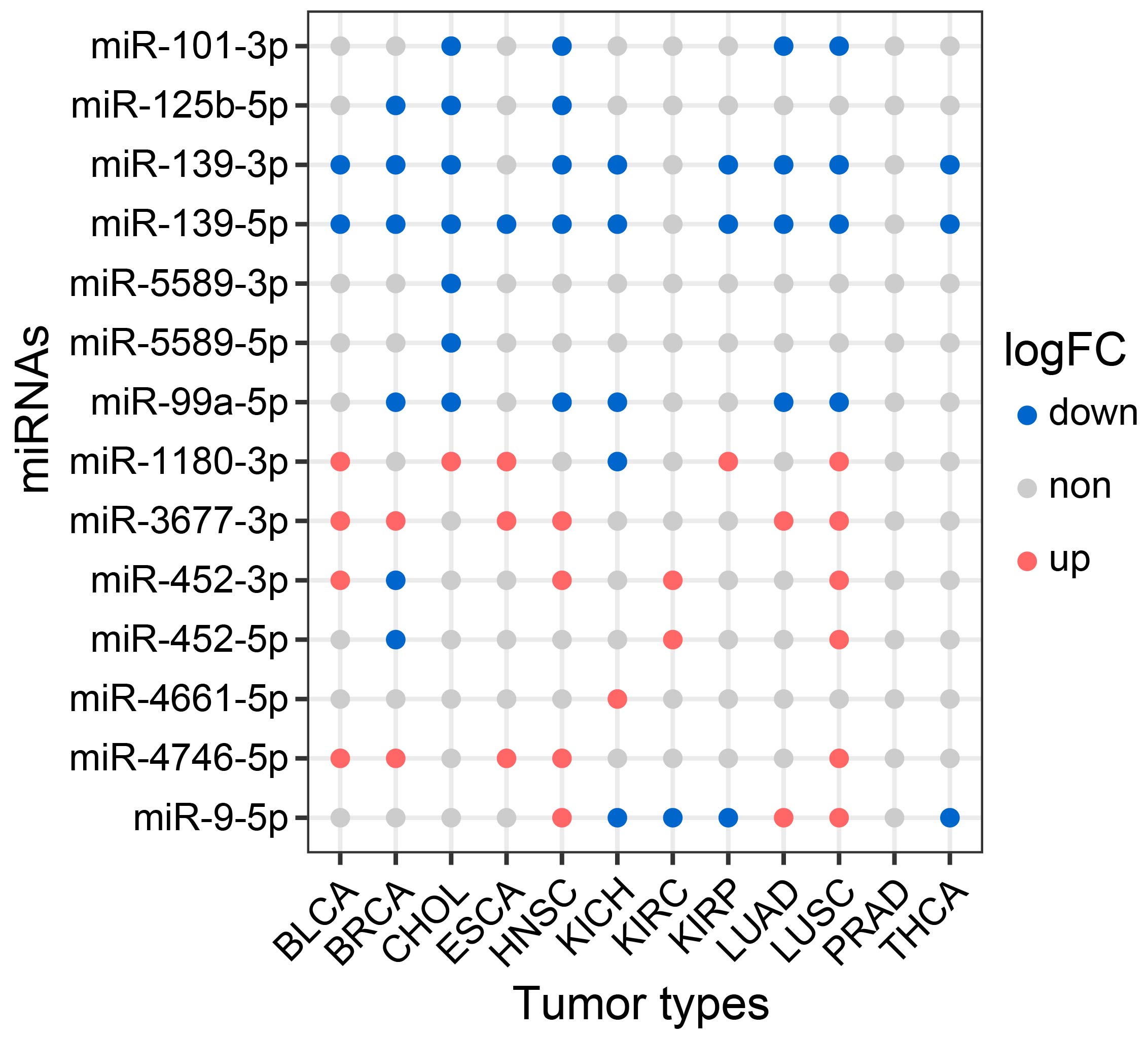

Supplement: Supplementary Figure 6 — The expression changes of 14 independent prognostic miRNAs in 12 other tumors types. Only matched tumor and adjacent tissues are used for calculation. The threshold of differentially expressed genes is |logFC| > 1 and FDR < 0.05. Red represents up-regulation, blue represents down-regulation, and gray represents no difference of gene expression. The full name of tumor types are as follows. BLCA, Bladder Urothelial Carcinoma; BRCA, Breast invasive carcinoma; CHOL, Cholangio carcinoma; ESCA, Esophageal carcinoma; HNSC, Head and Neck squamous cell carcinoma; KICH, Kidney Chromophobe; KIRC, Kidney renal clear cell carcinoma; KIRP, Kidney renal papillary cell carcinoma; LUAD, Lung adenocarcinoma; LUSC, Lung squamous cell carcinoma; PRAD, Prostate adenocarcinoma; THCA, Thyroid carcinoma. [file Image_6.jpeg]

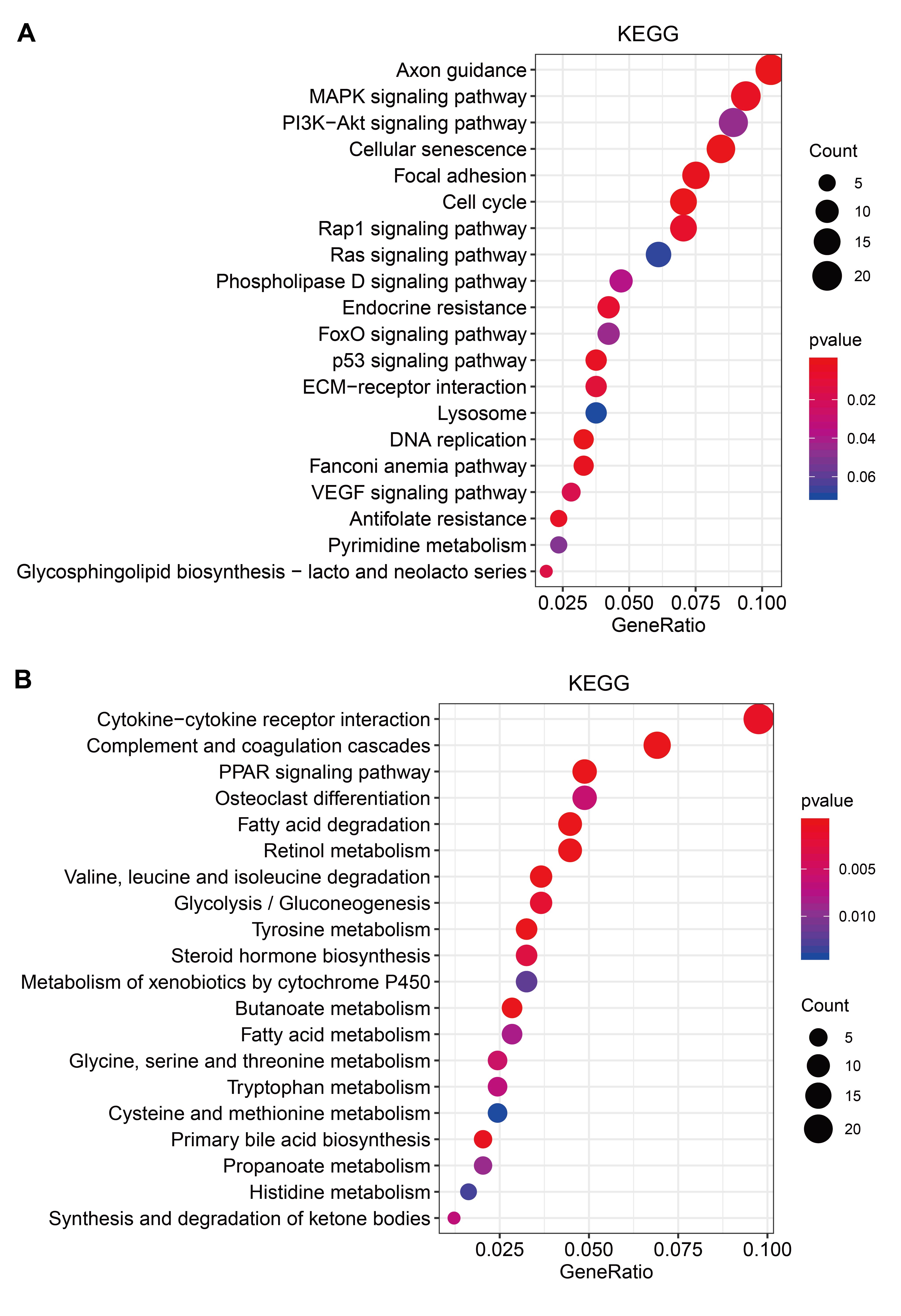

Supplement: Supplementary Figure 7 — KEGG pathway enrichment analysis of 14 prognostic miRNA target genes. (A) KEGG pathway enrichment analysis of up-regulated target genes. (B) KEGG pathway enrichment analysis of down-regulated target genes. [file Image_7.jpeg]

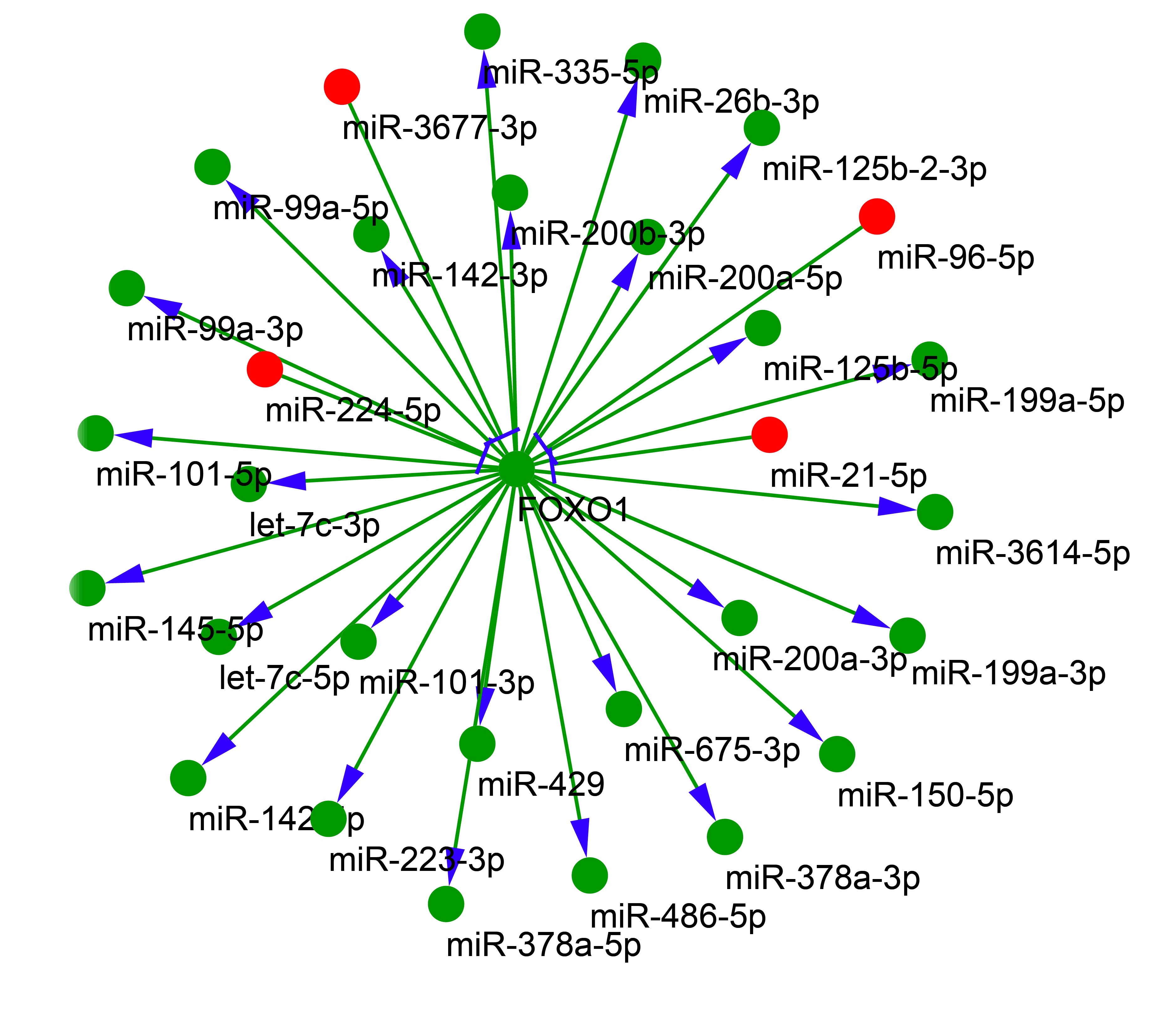

Supplement: Supplementary Figure 8 — Interaction between FOXO1 and miRNAs. Red represents up-regulation of expression, and green represents down-regulation of expression. [file Image_8.jpeg]

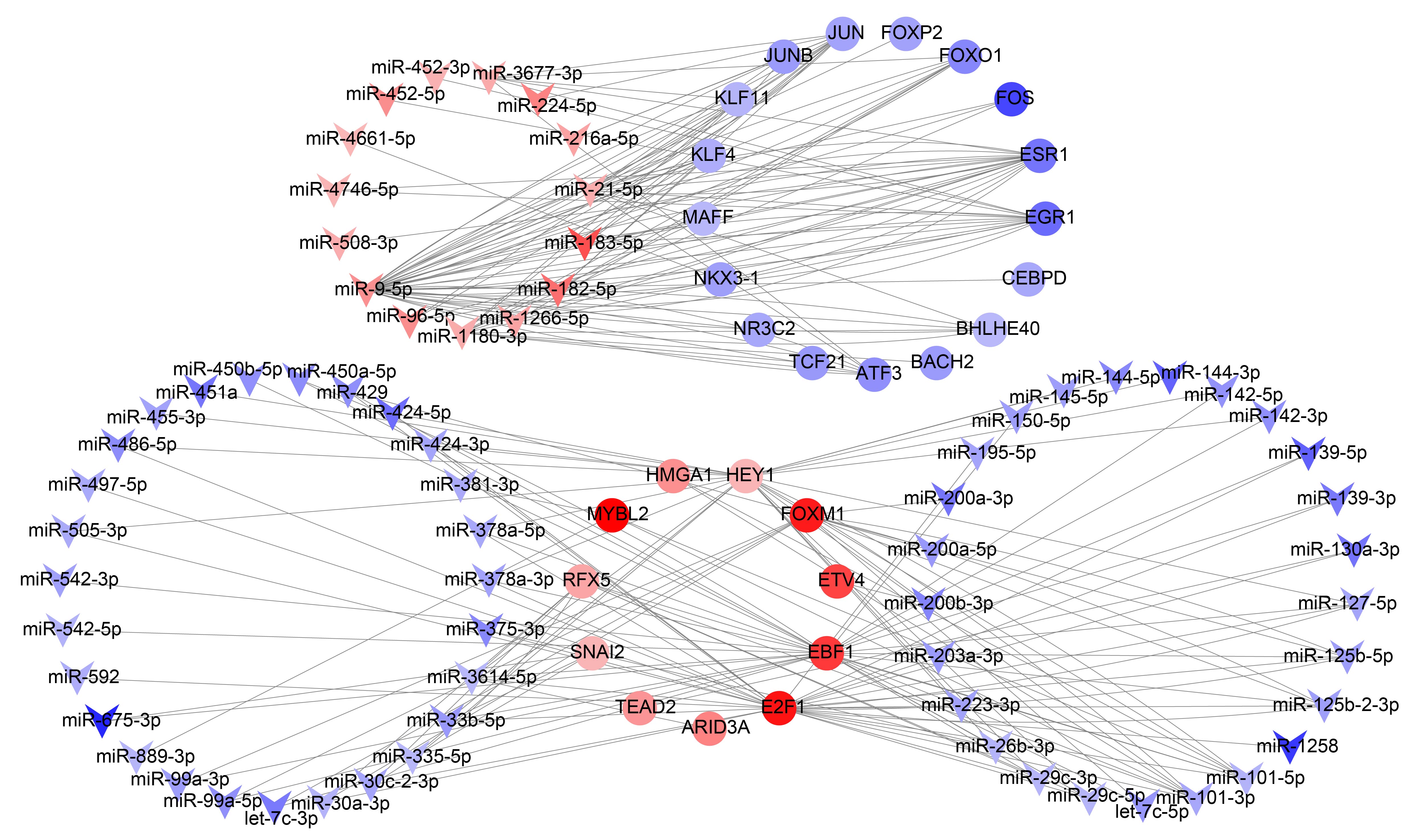

Supplement: Supplementary Figure 9 — Network diagram of predicted transcription factor suppression miRNA. The circles represent transcription factors, and the triangles represent miRNAs. Red represents up-regulation and blue represents down-regulation. [file Image_9.jpeg]
